# Does ICSI outcome in obstructive azoospermia differ according to the origin of retrieved spermatozoa or the cause of epididymal obstruction? A comparative study

**DOI:** 10.1007/s11255-022-03350-x

**Published:** 2022-09-05

**Authors:** Xiaochen Yu, Shaoming Lu, Mingzhen Yuan, Gang Ma, Xiao Li, Taijian Zhang, Shanshan Gao, Daimin Wei, Zi-Jiang Chen, Hongbin Liu, Haobo Zhang

**Affiliations:** 1 Center for Reproductive Medicine, Shandong University, Jinan, 250012 Shandong China; 2 Key Laboratory of Reproductive Endocrinology of Ministry of Education, Shandong University, Jinan, 250012 Shandong China; 3 Shandong Key Laboratory of Reproductive Medicine, Jinan, 250012 Shandong China; 4 Shandong Provincial Clinical Research Center for Reproductive Health, Jinan, 250012 Shandong China; 5 The Second Hospital, Cheeloo College of Medicine, Shandong University, Jinan, 250012 Shandong China; 6 Shanghai Key Laboratory for Assisted Reproduction and Reproductive Genetics, Shanghai, 200135 China

**Keywords:** Intracytoplasmic sperm injection, Obstructive azoospermia, Testicular sperm aspiration, Percutaneous epididymal sperm aspiration, Granulocyte elastase

## Abstract

**Purpose:**

To determine whether ICSI outcomes are affected by sperm source or genital tract inflammatory status.

**Methods:**

A retrospective cohort study was conducted in all consecutive obstructive azoospermia patients who underwent testicular sperm aspiration (TESA) or percutaneous epididymal sperm aspiration (PESA) and ICSI between February 1, 2017, and December 31, 2020. Couples were excluded if they were diagnosed with monogenic disease, abnormal karyotype, or had female uterine malformation. The primary objective was to determine whether ICSI outcomes are affected by the use of testicular or epididymal spermatozoa, and the secondary objective was to explore the effect of granulocyte elastase on ICSI outcomes using epididymal spermatozoa.

**Results:**

Compared with TESA, inflammatory and non-inflammatory PESA patients exhibited a better high-quality embryo rate, with significant differences among the three groups (49.43 vs. 55.39% and 56.03%; odds ratio, 6.345 and 6.631; 95% confidence interval, 0.340–12.350, and 1.712–11.550; *P* = 0.038 and *P* = 0.008, respectively). The fertilization rate, clinical pregnancy rate, live birth delivery rate, and congenital anomaly birth rate were similar in patients who underwent TESA or PESA (with or without inflammation).

**Conclusions:**

The high-quality embryo rate in PESA patients was higher than that in TESA patients. After successful pregnancy, ICSI outcomes did not differ between patients with obstructive azoospermia who experienced TESA or PESA and those with or without genital tract inflammation.

**Supplementary Information:**

The online version contains supplementary material available at 10.1007/s11255-022-03350-x.

## Introduction

Infertility affects approximately 15% of couples, and about 50% of infertility cases result from male factors [1–4]. The incidence of azoospermia is about 10–15% in male infertility cases [5, 6], and obstructive azoospermia (OA) accounts for almost 40% of azoospermia patients [7, 8]. OA is caused by a mechanical block along the reproductive tract, including the vas deferens, epididymis, and ejaculatory duct [9, 10]. The obstruction may be congenital or caused by trauma (surgical or non-surgical) or infection [9]. Normal spermatogenesis is characteristic of OA, and it is possible to restore reproductive function for OA patients via intracytoplasmic sperm injection (ICSI) using percutaneous epididymal sperm aspiration (PESA) or testicular sperm aspiration (TESA) [6].

PESA is a simple technique that can be performed under local anesthesia or mild sedation. For OA patients in whom sperm cannot be found in the epididymis, it is always possible to find sperm in the testis [11]. Both TESA and PESA have the advantages of no open surgical exploration, repeatability, minimal postoperative trauma, rapid process, and low cost [12]. The disulfide bond between protamines is formed during sperm transition from the caput epididymidis to the cauda epididymidis, and the sperm becomes mature with good physical appearance, motility, and fertilization ability [13]. PESA has the advantage of obtaining more mature sperm [12]. Also, the quality of spermatozoa from PESA may be altered because the distal epididymis contains a large number of sperm fragments with macrophages [9]. Percutaneous sperm retrievals are simple and effective methods for collecting epididymal or testicular spermatozoa in OA [9]. TESA should only be performed as a rescue procedure because TESA may have a higher risk of complications [6, 9, 14]. Whether the source of sperm affects ICSI outcomes in OA patients is still debatable, and studies from different reproductive centers have not been unanimous [13–15]. A systematic review and meta-analysis in 2019 collected previous studies and found that there were no significant differences in pregnancy or miscarriage rates between TESA and PESA treatment groups, although the fertilization rate was slightly higher using PESA and the implantation rate was numerically higher using TESA [16]. In addition, there is little evidence for the superiority of TESA or PESA, which is in accordance with a previous synthesis in the Cochrane Database [17].

Infections and the resulting inflammatory reactions in the male genital tract are among the main causes of male infertility, accounting for 6–10% of all cases [18]. Infection is indicated by an increased level of leukocytes (particularly polymorphonuclear leukocytes) and their products (e.g. leukocyte elastase) secreted into the seminal fluid [19]. Seminal plasma elastase is a biochemical indicator of polymorphonuclear lymphocyte activity in the ejaculate, with a suggested cut-off level of approximately 600 ng/ml [19]. Granulocyte elastase is a sensitive silent male genital tract inflammation indicator and indicates a state of inflammation with or without clinical symptoms [20–22]. Because granulocyte elastase reflects male genital tract inflammation, the testicular environment is not affected, so we divided our study population into TESA, PESA without genital tract inflammation, and PESA with genital tract inflammation. To simplify the grouping names, “non-inflammatory PESA” refers to PESA without genital tract inflammation, and “inflammatory PESA” refers to PESA with genital tract inflammation.

ICSI is an effective assisted reproductive technology in the treatment of OA in male infertility. However, the association between the origin of retrieved spermatozoa and the cause of epididymal obstruction and ICSI outcomes is uncertain. In this study, the primary objective was to determine whether ICSI outcomes are affected by TESA or PESA, and the secondary objective was to explore the effect of granulocyte elastase on ICSI outcomes from PESA.

## Materials and methods

### Study design and population

We retrospectively collected the records of consecutive OA patients after ICSI with TESA or PESA at the Reproductive Hospital of Shandong University, People’s Republic of China, from February 2017 to December 2020. Basic patient information and ICSI data were obtained through the hospital's medical record system, and neonatal outcomes were obtained and updated by the hospital's follow-up staff through regular telephone calls to the couples who had undergone assisted reproductive technology. The OA patients who underwent PESA were divided into the inflammation or non-inflammation group according to their seminal plasma granulocyte elastase level (< 600 ng/ml, non-inflammation; > 600 ng/ml, inflammation) [19]. Azoospermia was diagnosed when no spermatozoa were found in semen samples after centrifugation at 3000×*g* for 15 min, and each patient underwent semen analyses twice according to the WHO guidelines [23]. OA is the absence of spermatozoa and spermatogenetic cells in semen and post-ejaculate urine due to bilateral obstruction of the seminal ducts. The azoospermia patients with at least one normal-sized testis, normal endocrine parameters, and normal spermatogenic function confirmed by testicular histology were diagnosed as OA. Karyotyping analysis and physical examination were conducted for each patient to screen for chromosomal abnormalities and to measure sex hormone levels. To avoid possible sources of bias, only the first fresh embryo transfer cycle per couple was included [15]. In addition, only females aged between 20 and 40 years and only couples who underwent ICSI with the husband’s fresh sperm were included in this study. Couples were excluded if they were diagnosed with monogenic disease, abnormal karyotype, or female uterine malformation. This study examined 240 patients who underwent TESA, 401 patients who underwent non-inflammatory PESA, and 158 patients who underwent inflammatory PESA. This study was approved and granted consent exemption by the Reproductive Hospital of Shandong University (IRB #2017-177). All procedures were performed in accordance with the ethical standards of the institutional research committee and the principles of the Declaration of Helsinki.

### Elastase assay

The elastase concentration in seminal plasma was measured as previously described [21, 24] using a commercial ELISA kit for the determination of the PMN-Elastase level in seminal plasma (Bo Rui de Biological Technology Co., LTD, Shenzhen, China). The principle of the technique is that granulocyte elastase is often in the form of its complex with the α1-protease inhibitor Ela/α1–PI. Elastase was captured by micropores precoated with elastase primary antibodies. After washing, HRP-labeled specific secondary antibody was added. Then, citric acid buffer containing 0.4625 g/L sodium perborate and 30% hydrogen peroxide was added as a colorigenic substrate, after which the absorbances were detected. The concentration of the fully formed elastase-α1-PI complex was positively correlated with the color intensity of the substrate.

### Preparation of sperm and ICSI

The TESA and PESA procedures were performed as described previously [23, 25]. Briefly, under local anesthesia a butterfly needle was passed into the scrotum with a fixed syringe filled with 1 mL sperm wash medium. Vacuum in the tube of the butterfly needle was maintained by suction from the syringe, and a hemostat was set. The needle was passed into the caput epididymidis, after which the hemostat was released and spermatozoa moved into the tube under vacuum. The samples were analyzed under an optical microscope. OA patients underwent PESA first. When no spermatozoa were obtained or the spermatozoa were of poor quality, such as immobility or severe deformity, TESA was performed as a rescue procedure with similar procedures to obtain spermatozoa from the testis.

The ICSI procedure was performed as previously described [26, 27]. In summary, routine stimulation was adopted according to the patients’ partners’ case requirements [28]. Oocyte retrieval was performed 34–36 h after human chorionic gonadotrophin injection, and luteal phase support was started and continued until 10 weeks of gestation. Cleaved embryos on day 3 of embryo culture or blastocysts on day 5 were transferred according to the scores by checking cell number, fragmentation, and cell symmetry [29, 30].

### Outcome measures

In our study, the primary outcome measures were clinical pregnancy rate and live birth delivery rate (LBDR). The secondary outcome measures included fertilization rate, high-quality embryo rate (HQER), implantation rate, no embryo suitable for transfer cycles rate, miscarriage rate, birth weight, and congenital anomaly birth rate. The calculations of ICSI outcomes were as described previously [29]. The biochemical pregnancy rate was defined as the number of biochemical pregnancies divided by the number of embryo transfer cycles, the clinical pregnancy rate was defined as the number of clinical pregnancies divided by the number of embryo transfer cycles, and LBDR was defined as the percentage of live birth delivery cycles out of the total number of embryo transfer cycles. All live births were included regardless of gestational age or the occurrence of twin pregnancy [31].

### Statistical analysis

All data analyses were performed using SPSS version 21.0 (IBM, Inc.). Categorical variables were expressed as frequencies and percentages, and Pearson’s chi-square or Fisher’s exact tests were used to compare the three groups. Continuous variables were tested for normality by the Shapiro–Wilk test. When continuous variables were normally distributed, they are presented as the mean ± standard deviation, and when continuous variables were not normally distributed, they are presented as the median and interquartile range (IQR). Differences were compared by one-way analysis of variance (ANOVA) or Welch’s ANOVA. Bonferroni correction was applied for three-group comparisons of continuous data. *P* < 0.05 was considered to be statistically significant. Differences and similarities among the three study groups were confirmed by linear regression for continuous data and by logistic regression for categorical data. Adjustments for regression included male age, male body mass index (BMI), male follicle-stimulating hormone (FSH), male luteinizing hormone (LH), male testosterone (T), male absence of dysplasia of seminal vesicles, female age, female BMI, female polycystic ovary syndrome (PCOS), female intrauterine adhesion, and oocytes retrieved < 5, according to previous studies [29, 31]. The regression results are presented separately as the unadjusted beta coefficient/odds ratio (u-*β*/OR), which only included the grouping variable, and the adjusted beta coefficient/odds ratio (a-*β*/OR), which contained all variables described above simultaneously, with the 95% confidence interval (CI). Goodness-of-fit was used for logistic regression models with statistical significance using the Hosmer–Lemeshow test.

## Results

### Clinical characteristics of patients

We identified 799 men who met the inclusion criteria and did not meet the exclusion criteria of this study, including 240 men who underwent TESA, 401 men who underwent PESA without genital tract inflammation, and 158 men who underwent PESA with genital tract inflammation. The demographic and clinical characteristics of the couples are presented in Table 1. No statistically significant differences were observed among the three groups with respect to male T, female PCOS, or female intrauterine adhesion. There were statistically significant differences among the three groups for female age, female BMI, male age, male BMI, male FSH, male LH, and causes of OA.

**Table 1:** Clinical characteristics of couples with male patients undergoing TESA or PESA

	TESA (*n* = 240)	Non-inflammatory PESA (*n* = 401)	Inflammatory PESA (*n* = 158)	*P* value
Female age (years)^e^	30.20 ± 4.53	28.93 ± 4.35	29.71 ± 4.45	**0.001**^**a**^
Female BMI (kg/m^2^)^e^	24.03 ± 4.01	23.15 ± 3.50	23.75 ± 3.19	**0.011**^**b**^
Male age (years)^e^	31.28 ± 5.62	30.22 ± 5.22	31.14 ± 5.48	**0.031**^**a**^
Male BMI (kg/m^2^)^e^	26.40 ± 4.30	25.55 ± 3.71	25.70 ± 3.77	**0.037**^**b**^
Male FSH (IU/L)^e,f^	4.98 (3.58, 7.48)	4.11 (3.01, 5.96)	4.07 (3.10, 5.32)	** < 0.001**^**b**^
Male LH (IU/L)^e^	4.56 (3.50, 6.33)	4.21 (3.26, 5.58)	4.61 (3.23, 5.97)	**0.033**^**a**^
Male T (ng/dL)	387.00 (305.00, 516.52)	396.40 (312.50, 523.30)	415.00 (314.30, 542.50)	0.542^a^
Female PCOS, % (n)	20.42 (49/240)	23.19 (93/401)	21.52 (34/158)	0.704^c^
Female intrauterine adhesion, % (*n*)	2.08 (5/240)	4.49 (18/401)	1.27 (2/158)	0.088^d^
Causes of OA				
Absence or dysplasia of seminal vesicles, % (*n*)^e,f^	9.58 (23/240)	22.69 (91/401)	19.62 (31/158)	** < 0.001**^**c**^
Surgery, % (*n*)	6.25 (15/240)	2.49 (10/401)	2.53 (4/158)	**0.034**^**c**^
Other factors, % (*n*)^e^	84.17 (202/240)	74.81 (300/401)	77.85 (123/158)	**0.021**^**c**^

Results are presented as the mean ± standard deviation, median and IQR, or % (*n*)

*IQR* interquartile ranges, *TESA* testicular sperm aspiration, *PESA* percutaneous epididymal sperm aspiration, *ANOVA* analysis of variance, *BMI* body mass index, *FSH* follicle-stimulating hormone, *LH* luteinizing hormone, *T* testosterone, *PCOS* polycystic ovary syndrome

*P* values in bold are statistically significant (<0.05)

^a^One-way ANOVA

^b^Welch’s ANOVA

^c^Pearson Chi-square test

^d^Fisher's exact test

^e^Statistically significant differences between TESA and non-inflammatory PESA

^f^Statistically significant differences between TESA and inflammatory PESA

The obstruction can be caused by many factors such as infection, surgery, trauma, or congenital factors [9, 11]. We used granulocyte elastase to indicate genital tract infection, which does not affect the testicular environment. Because patients who underwent PESA had been divided into the inflammatory and non-inflammatory groups, we did not include the frequency or numbers of infection factors in OA causes. Ligation of the vas deferens, vasectomy, inguinal hernia, or improper cutting or ligation of the vas deferens during undescended testicle surgery were summarized as surgery. Congenital defects often include the absence of the vas deferens accompanied by epididymal hypoplasia and/or abnormal or absent seminal vesicle glands [32], so we used seminal vesicles to indicate congenital defects. Epididymal cysts and unexplained vas deferens obstruction were attributed to other factors.

### Embryologic outcomes of the study population

The embryologic outcomes are shown in Table 2 and Table S1. The HQER of men with TESA was lower than in men with inflammatory or non-inflammatory PESA (49.43% vs. 55.39% and 56.03%, respectively; adjusted odds ratio (OR), 6.345 and 6.631; 95% confidence interval (CI), 0.340–12.350, and 1.712–11.550; *P* = 0.038 and *P* = 0.008, respectively). There were no significant differences among the three groups for oocyte number retrieved, fertilization rate, or 2PN cleavage rate.

**Table 2:** Embryologic outcomes of male patients with TESA or PESA

	TESA (*n* = 240)	Non-inflammatory PESA (*n* = 401)	Inflammatory PESA (*n* = 158)	*P* value
Oocytes retrieved < 5, % (*n*)	7.50 (18/240)	8.73 (35/401)	8.86 (14/158)	0.838^b^
Oocytes retrieved	11 (8, 15)	12 (8, 16)	12 (8, 16)	0.092^a^
Metaphase II oocytes	10 (7, 12)	10 (7, 13)	10 (7, 13)	0.286^a^
Fertilized oocytes (2PN)	7 (5, 9)	7 (5, 10)	7 (4.5, 10)	0.264^a^
FR, % (*n*)	72.19 (1659/2298)	72.25 (2927/4051)	73.83 (1179/1597)	0.442^b^
2PN cleavage zygotes	7 (4.75, 9)	7 (5, 10)	7 (4.5, 10)	0.273^a^
2PNCR, % (*n*)	99.16 (1645/1659)	99.49 (2912/2927)	99.32 (1171/1179)	0.396^b^
High-quality embryo^c,d^	3 (2, 5)	4 (2, 6)	4 (2, 6)	**0.004**^**a**^
HQER, % (*n*)^c,d^	49.43 (820/1659)	56.03 (1640/2927)	55.39 (653/1179)	** < 0.001**^**b**^

Results are presented as the median and interquartile ranges, or % (*n*)

*TESA* testicular sperm aspiration, *PESA* percutaneous epididymal sperm aspiration, *ANOVA* analysis of variance, *FR* fertilization rate, *2PNCR* 2PN cleavage rate, *HQER* high-quality embryo rate

*P* values in bold are statistically significant (<0.05)

^a^One-way ANOVA

^b^Pearson Chi-square test

^c^Statistically significant differences between TESA and non-inflammatory PESA

^d^Statistically significant differences between TESA and inflammatory PESA

### Embryo transfer outcomes of patients

The embryo transfer outcomes are shown in Table 3 and Table S1. The percentage of cycles cancelled due to no high-quality embryos was higher for men with TESA than for men with non-inflammatory PESA (21.43% vs. 9.26%; adjusted OR, 0.372; 95% CI, 0.149–0.927; *P* = 0.034). The miscarriage rate was lower for men with inflammatory PESA than for men with TESA (1.89% vs. 11.11%; adjusted OR, 0.118; 95% CI, 0.014–0.971; *P* = 0.047), and the logistic regression model fitness (Hosmer–Lemeshow) showed that the model fit the data with a *p* value of 0.642. There were no significant differences among the three groups for implantation rate, no embryo suitable for transfer cycles’ rate, biochemical pregnancy rate, clinical pregnancy rate, ectopic pregnancy rate, singleton or twin pregnancy rate, or LBDR.

**Table 3:** Embryo transfer parameters of male patients with TESA or PESA

	TESA (*n* = 240)	Non-inflammatory PESA (*n* = 401)	Inflammatory PESA (*n* = 158)	*P* value
No. of embryos transferred	2 (2, 2)	2 (2, 2)	2 (1.5, 2)	0.577^a^
No. of gestational sacs	1 (0, 1)	1 (0, 2)	1 (0, 1)	0.514^a^
IR, % (*n*)	46.69 (127/272)	49.65 (210/423)	44.12 (75/170)	0.444^b^
NESTCR, % (*n*)	35.00 (84/240)	40.40 (162/401)	37.97 (60/158)	0.394^b^
Reason for not undergoing embryo transfer				
Frozen embryos, % (*n*)	8.33 (7/84)	4.94 (8/162)	8.33 (5/60)	0.433^c^
Female factors, % (*n*)^d^	58.33 (49/84)	76.54 (124/162)	76.67 (46/60)	**0.007**^**b**^
No high-quality embryos, % (*n*)^d^	21.43 (18/84)	9.26 (15/162)	13.33 (8/60)	**0.029**^**b**^
Others factors, % (*n*)	11.90 (10/84)	9.26 (15/162)	1.67 (1/60)	0.083^b^
Biochemical pregnancy, % (*n*)	7.05 (11/156)	6.28 (15/239)	11.22 (11/98)	0.284^b^
Clinical pregnancy, % (*n*)	57.69 (90/156)	62.76 (150/239)	54.08 (53/98)	0.293^b^
Ectopic pregnancy, % (*n*)	0.00 (0/156)	1.26 (3/239)	1.02 (1/98)	0.419^c^
SPR, % (*n*)	58.89 (53/90)	56.67 (85/150)	54.72 (29/53)	0.882^b^
TPR, % (*n*)	41.11 (37/90)	41.33 (62/150)	43.40 (23/53)	0.959^b^
LBDR, % (*n*)	51.28 (80/156)	57.74 (138/239)	52.04 (51/98)	0.395^b^
MR, % (*n*)	11.11 (10/90)	6.00 (9/150)	1.89 (1/53)	0.104^c^

Results are presented as the median and interquartile ranges, or % (*n*)

*TESA* testicular sperm aspiration, *PESA* percutaneous epididymal sperm aspiration, *ANOVA* analysis of variance, *IR* implantation rate, *NESTCR* no embryo suitable for transfer cycles rate, *SPR* singleton pregnancy rate, *TPR* twin pregnancy rate, *LBDR* live birth delivery rate *MR* miscarriage rate

*P* values in bold are statistically significant (<0.05)

^a^One-way ANOVA

^b^Pearson Chi-square test

^c^Fisher’s exact test

^d^Statistically significant differences between TESA and non-inflammatory PESA

### Newborn outcomes among the three groups

The newborn outcomes are shown in Table 4 and Table S1. The birth weight of twin newborns was higher for men who underwent PESA (with or without inflammation) than men with TESA (2.76 ± 0.33 kg and 2.57 ± 0.44 kg vs. 2.39 ± 0.63 kg; adjusted OR, 0.487 and 0.301; 95% CI, 0.246–0.727 and 0.103–0.499; *P* < 0.001 and *P* = 0.003, respectively). There were no significant differences among the three groups for term and preterm LBDR, singleton or twin LBDR, gestational age, birth weight of singleton newborns, or congenital anomaly birth rate.

**Table 4:** Newborn outcomes of male patients with TESA or PESA

	TESA (*n* = 240)	Non-inflammatory PESA (*n* = 401)	Inflammatory PESA (*n* = 158)	*P* value
Term LBDR, % (*n*)	83.75 (67/80)	79.71 (110/138)	82.35 (42/51)	0.747^c^
Preterm LBDR, % (*n*)	16.25 (13/80)	20.29 (28/138)	17.65 (9/51)	0.747^c^
Singleton LBDR, % (*n*)	65.00 (52/80)	62.32 (86/138)	74.51 (38/51)	0.293^c^
Twin LBDR, % (*n*)	35.00 (28/80)	37.68 (52/138)	25.49 (13/51)	0.293^c^
Gestational age (days)	266.83 ± 19.9	268.51 ± 14.36	269.82 ± 12.84	0.558^a^
Birth weight (kg)				
Singleton				
Mean ± SD	3.34 ± 0.49	3.3 ± 0.58	3.34 ± 0.58	0.912^a^
No. of observations	52	86	38	
Twin				
Mean ± SD^e,f^	2.39 ± 0.63	2.57 ± 0.44	2.76 ± 0.33	**0.005**^**b**^
No. of observations	56	102	26	
Congenital anomaly birth				
Congenital heart defects, % (*n*)	1.85 (2/108)	1.05 (2/190)	0.00 (0/64)	0.651^d^
Other abnormalities, % (*n*)	1.85 (2/108)	1.58 (3/190)	0.00 (0/64)	0.714^d^

Results are presented as the mean ± standard deviation or % (*n*)

*TESA* testicular sperm aspiration, *PESA* percutaneous epididymal sperm aspiration, *ANOVA* analysis of variance, *LBDR* live birth delivery rate, *SD* standard deviation

*P* values in bold are statistically significant (<0.05)

^a^One-way ANOVA

^b^Welch’s ANOVA

^c^Pearson Chi-square test

^d^Fisher's exact test

^e^Statistically significant differences between TESA and inflammatory PESA

^f^Statistically significant differences between non-inflammatory PESA and inflammatory PESA

## Discussion

This study assessed the effect of both the source of sperm and genital tract inflammation factors on ICSI outcomes in OA patients. We found that sperm aspiration from the testis adversely affected the HQER compared with sperm aspiration from the epididymis and that the presence of inflammation in PESA did not affect ICSI outcomes.

Previous studies have reported that OA patients undergoing PESA achieve comparable ICSI outcomes as those undergoing TESA, with a higher fertilization rate and lower miscarriage rate but with similar pregnancy and live birth rates [14, 33–35]. In our study, compared with patients with TESA, patients with PESA had a higher HQER and birth weight of twin newborns (regardless of inflammation status). Also, the miscarriage rate in the PESA group was lower than that of the TESA group, although only the difference between the PESA patients with inflammation and the TESA patients was statistically significant. The fertilization rate, pregnancy rate, and LBDR were similar among the three groups.

Although there was a significant difference in female and male age among the three groups in the current study, the age of the females and males in none of the three groups was advanced enough to deleteriously impact ICSI outcome (*i.e.* female age > 40 and male age > 50) [36, 37]. Also, despite the significant differences among the three groups for male FSH and LH, the values for all three groups were within the normal ranges and did not reach levels known to affect assisted reproductive technology outcomes [38]. Previous studies have reported that inflammation and congenital factors may influence ICSI outcomes [39, 40], while other factors such as surgery do not affect ICSI outcomes [10, 15, 31]. Therefore, while there were significant differences in female and male BMI and causes of OA, we contained BMI and congenital factors (the absence or dysplasia of seminal vesicles) in our linear and logistic regression analyses in order to manage possible bias [29, 32].

The HQER for the PESA group, regardless of inflammation status, was higher than for the TESA group. In correspondence with the HQER, the rate of not undergoing embryo transfer due to lack of high-quality embryos in the TESA group was higher than that of PESA group. These results indicated that sperm from the epididymis apparently have better developmental potential than sperm from the testis. A plausible explanation for this observed difference could be that immature testicular spermatozoa in OA patients may involve genomic imprinting, which impairs the embryologic outcomes compared to mature sperm from the epididymis [35, 41].

Previous studies showed different fertilization rates and miscarriage rates between TESA and PESA [14, 33–35]. A systematic review in 2019 gathered previous study data and found that there were no significant differences in pregnancy or miscarriage rates between the TESA and PESA treatment groups, although the fertilization rate was slightly higher using PESA and the implantation rate was numerically higher using TESA [16]. In our study, the fertilization rate, 2PN cleavage rate, implantation rate, no embryo suitable for transfer cycles rate, biochemical pregnancy rate, clinical pregnancy rate, ectopic pregnancy rate, singleton pregnancy rate, and twin pregnancy rate did not differ among the three groups. The LBDR in the TESA group was slightly lower than that in PESA groups, but the difference was not statistically significant. In accordance with previous research, the miscarriage rate in the TESA group was higher than in the PESA groups, which may be attributed to the more mature sperm from the epididymides compared to the testes. Although the difference between the inflammatory PESA patients and the TESA patients was statistically significant (*P* = 0.047), the sample size was not large enough to conclude that the miscarriage rate favored inflammatory PESA over TESA. For an α-error of 0.05 and a power of 80%, the calculated size of inflammatory PESA patients was 71, which was more than the 53 included in our study.

Previous studies have reported that neonatal outcomes and congenital malformations in children born after ICSI with testicular or epididymal sperm are similar [6, 42]. Consistent with these studies, our data support that term and preterm LBDR, singleton and twin LBDR, gestational age, birth weight of singleton newborns, and congenital anomaly birth rate were similar among the three examined groups. We found that the birth weight of twin newborns was statistically higher in PESA patients than TESA patients regardless of whether PESA was performed in patients with inflammation. This result can perhaps be explained by our data showing a younger gestational age for the TESA patients compared to the PESA patients, although the difference was not statistically significant.

An innovation of the present study was our analysis of whether reproductive tract inflammation (assessed based on elastase levels) might impact ICSI outcomes, and our data indicate that genital tract inflammation does not obviously affect ICSI outcomes. However, there are two limitations of our study. First, there are many other ways to assess inflammation, such as leukocytes (< 1 × 10^6^/ml) and proinflammatory cytokines (e.g. IL-6 [30 pg/mL] and IL-8 [7000 pg/mL]) [43]. Second, although we took into consideration female covariates like PCOS, intrauterine adhesion, and ovarian-poor responders (oocytes retrieved < 5), some female factors known to affect ICSI outcomes were not considered in our study such as hormone level and endometrial thickness [27, 29]. These factors may cause possible residual confounding.

In conclusion, our results indicate that the sperm from TESA or PESA did not affect ICSI outcomes such as pregnancy rate and live birth delivery rate, although the TESA procedure had a negative effect on the embryonic outcome of HQER in OA patients. Moreover, male genital inflammation as determined by granulocyte elastase levels did not affect ICSI outcomes. Our results provide referential experience for counseling OA patients in ICSI outcomes and potential treatments.

## Supplementary Information

Below is the link to the electronic supplementary material.

Supplementary file1 (XLSX 16 KB)

## References

[1] de Kretser DM (1997). Male infertility. Lancet.

[2] Agarwal A, Mulgund A, Hamada A, Chyatte MR (2015). A unique view on male infertility around the globe. Reprod Biol Endocrinol.

[3] Di Spiezio Sardo A, Di Carlo C, Minozzi S, Spinelli M, Pistotti V, Alviggi C, De Placido G, Nappi C, Bifulco G (2016). Efficacy of hysteroscopy in improving reproductive outcomes of infertile couples: a systematic review and meta-analysis. Hum Reprod Update.

[4] Ghieh F, Mitchell V, Mandon-Pepin B, Vialard F (2019). Genetic defects in human azoospermia. Basic Clin Androl.

[5] Plaseska-Karanfilska D, Noveski P, Plaseski T, Maleva I, Madjunkova S, Moneva Z (2012). Genetic causes of male infertility. Balkan J Med Genet.

[6] Jin L, Li Z, Gu L, Huang B (2020). Neonatal outcome of children born after ICSI with epididymal or testicular sperm: a 10-year study in China. Sci Rep.

[7] Dong M, Li H, Zhang X, Tan J (2021). Weighted correlation gene network analysis reveals new potential mechanisms and biomarkers in non-obstructive azoospermia. Front Genet.

[8] Practice Committee of the American Society for Reproductive Medicine in collaboration with the Society for Male R Urology (2019). The management of obstructive azoospermia: a committee opinion. Fertil Steril.

[9] Miyaoka R, Esteves SC (2013). Predictive factors for sperm retrieval and sperm injection outcomes in obstructive azoospermia: do etiology, retrieval techniques and gamete source play a role?. Clinics.

[10] Esteves SC, Lee W, Benjamin DJ, Seol B, Verza S, Agarwal A (2013). Reproductive potential of men with obstructive azoospermia undergoing percutaneous sperm retrieval and intracytoplasmic sperm injection according to the cause of obstruction. J Urol.

[11] Popal W, Nagy ZP (2013). Laboratory processing and intracytoplasmic sperm injection using epididymal and testicular spermatozoa: what can be done to improve outcomes?. Clinics.

[12] Esteves SC, Miyaoka R, Orosz JE, Agarwal A (2013). An update on sperm retrieval techniques for azoospermic males. Clinics.

[13] Dozortsev D, Neme R, Diamond MP, Abdelmassih S, Abdelmassih V, Oliveira F, Abdelmassih R (2006). Embryos generated using testicular spermatozoa have higher developmental potential than those obtained using epididymal spermatozoa in men with obstructive azoospermia. Fertil Steril.

[14] Borges E, Rossi-Ferragut LM, Pasqualotto FF, dos Santos DR, Rocha CC, Iaconelli A (2002). Testicular sperm results in elevated miscarriage rates compared to epididymal sperm in azoospermic patients. Sao Paulo Med J.

[15] Kamal A, Fahmy I, Mansour R, Serour G, Aboulghar M, Ramos L, Kremer J (2010). Does the outcome of ICSI in cases of obstructive azoospermia depend on the origin of the retrieved spermatozoa or the cause of obstruction? A comparative analysis. Fertil Steril.

[16] Shih KW, Shen PY, Wu CC, Kang YN (2019). Testicular versus percutaneous epididymal sperm aspiration for patients with obstructive azoospermia: a systematic review and meta-analysis. Transl Androl Urol.

[17] Van Peperstraten A, Proctor ML, Johnson NP, Philipson G (2008). Techniques for surgical retrieval of sperm prior to intra-cytoplasmic sperm injection (ICSI) for azoospermia. Cochrane Database Syst Rev.

[18] Pilatz A, Boecker M, Schuppe HC, Diemer T, Wagenlehner F (2016). Infection and infertility. Urologe A.

[19] Jungwirth A, Diemer T, Dohle GR, Giwercman A, Kopa Z, Tournaye H, Krausz C (2013) Guidelines on Male Infertility. EAU, p 44 10.1016/j.eururo.2012.04.048 22591628

[20] Kopa Z, Wenzel J, Papp GK, Haidl G (2005). Role of granulocyte elastase and interleukin-6 in the diagnosis of male genital tract inflammation. Andrologia.

[21] Zorn B, Virant-Klun I, Meden-Vrtovec H (2000). Semen granulocyte elastase: its relevance for the diagnosis and prognosis of silent genital tract inflammation. Hum Reprod.

[22] Jochum M, Pabst W, Schill WB (1986). Granulocyte elastase as a sensitive diagnostic parameter of silent male genital tract inflammation. Andrologia.

[23] World Health Organization (2010). Laboratory manual for the examination and processing of human semen.

[24] Wolff H, Bezold G, Zebhauser M, Meurer M (1991). Impact of clinically silent inflammation on male genital tract organs as reflected by biochemical markers in semen. J Androl.

[25] Javed A, Ramaiah MK, Talkad MS (2019). ICSI using fresh and frozen PESA-TESA spermatozoa to examine assisted reproductive outcome retrospectively. Obstet Gynecol Sci.

[26] Wei D, Liu JY, Sun Y, Shi Y, Zhang B, Liu JQ, Tan J, Liang X, Cao Y, Wang Z (2019). Frozen versus fresh single blastocyst transfer in ovulatory women: a multicentre, randomised controlled trial. Lancet.

[27] Guo Z, Xu X, Zhang L, Zhang L, Yan L, Ma J (2020). Endometrial thickness is associated with incidence of small-for-gestational-age infants in fresh in vitro fertilization-intracytoplasmic sperm injection and embryo transfer cycles. Fertil Steril.

[28] Fang J, Shu L, Cai L, Cui Y, Liu J, Yang X (2020). Intracytoplasmic sperm injection outcomes in patients with orgasmic dysfunction and anejaculation by percutaneous epididymal sperm aspiration (PESA). Ann Transl Med.

[29] Zhang L, Mao JM, Li M, Lian Y, Lin SL, Chen LX, Yan LY, Qiao J, Liu P (2021). Poor intracytoplasmic sperm injection outcome in infertile males with azoospermia factor c microdeletions. Fertil Steril.

[30] Racowsky C, Stern JE, Gibbons WE, Behr B, Pomeroy KO, Biggers JD (2011). National collection of embryo morphology data into society for assisted reproductive technology clinic outcomes reporting system: associations among day 3 cell number, fragmentation and blastomere asymmetry, and live birth rate. Fertil Steril.

[31] Lopes LS, Cury VN, Cha JD, Lampa Junior VM, Marques JL, Mizrahi FE, Figueiredo F, Barbosa CP, Glina S (2020). Do assisted reproduction outcomes differ according to aetiology of obstructive azoospermia?. Andrologia.

[32] Rathaus V, Werner M, Freud E, Mei-Zahav M, Mussaffi H, Blau H (2006). Sonographic findings of the genital tract in boys with cystic fibrosis. Pediatr Radiol.

[33] Zhan XX, Wan CC, Li HB, Gou J, Cai HC, Zhao J, Yan CF, Diao ZY, Shang XJ (2016). ICSI with testicular or epididymal sperm for patients with obstructive azoospermia: a systematic review. Zhonghua Nan Ke Xue.

[34] Buffat C, Patrat C, Merlet F, Guibert J, Epelboin S, Thiounn N, Vieillefond A, Adda-Lievin A, Lebon C, Jouannet P (2006). ICSI outcomes in obstructive azoospermia: influence of the origin of surgically retrieved spermatozoa and the cause of obstruction. Hum Reprod.

[35] Semiao-Francisco L, Braga DP, Figueira Rde C, Madaschi C, Pasqualotto FF, Iaconelli A, Borges E (2010). Assisted reproductive technology outcomes in azoospermic men: 10 years of experience with surgical sperm retrieval. Aging Male.

[36] La Marca A, Capuzzo M, Imbrogno MG, Donno V, Spedicato G, Sacchi S, Minasi MG, Spinella F, Greco P, Fiorentino F, Greco E (2021). The complex relationship between female age and embryo euploidy. Minerva Obstet Gynecol.

[37] Horta F, Vollenhoven B, Healey M, Busija L, Catt S, Temple-Smith P (2019). Male ageing is negatively associated with the chance of live birth in IVF/ICSI cycles for idiopathic infertility. Hum Reprod.

[38] Liu Z, Shi X, Wang L, Yang Y, Fu Q, Tao M (2017). Biosci Rep.

[39] Nicopoullos JD, Gilling-Smith C, Ramsay JW (2004). Does the cause of obstructive azoospermia affect the outcome of intracytoplasmic sperm injection: a meta-analysis. BJU Int.

[40] Lu S, Cui Y, Li X, Zhang H, Liu J, Kong B, Cai F, Chen ZJ (2014). Association of cystic fibrosis transmembrane-conductance regulator gene mutation with negative outcome of intracytoplasmic sperm injection pregnancy in cases of congenital bilateral absence of vas deferens. Fertil Steril.

[41] Palermo GD, Schlegel PN, Colombero LT, Zaninovic N, Moy F, Rosenwaks Z (1996). Aggressive sperm immobilization prior to intracytoplasmic sperm injection with immature spermatozoa improves fertilization and pregnancy rates. Hum Reprod.

[42] Fedder J, Loft A, Parner ET, Rasmussen S, Pinborg A (2013). Neonatal outcome and congenital malformations in children born after ICSI with testicular or epididymal sperm: a controlled national cohort study. Hum Reprod.

[43] Schuppe HC, Pilatz A, Hossain H, Diemer T, Wagenlehner F, Weidner W (2017). Urogenital infection as a risk factor for male infertility. Dtsch Arztebl Int.

